# The effect of socioeconomic status on informal caregiving for parents among adult married females: evidence from China

**DOI:** 10.1186/s12877-021-02094-0

**Published:** 2021-03-06

**Authors:** Yi Wang, Jiajia Li, Lulu Ding, Yuejing Feng, Xue Tang, Long Sun, Chengchao Zhou

**Affiliations:** 1 Centre for Health Management and Policy Research, School of Public Health, Cheeloo College of Medicine, Shandong University, Jinan, 250012 China; 2 NHC Key Laboratory of Health Economics and Policy Research, Shandong University, 44 Wen-hua-xi Road, Jinan, 250012 Shandong China

**Keywords:** Informal caregiving, Socioeconomic status, Married females, China

## Abstract

**Background:**

Married female caregivers face a higher risk of an informal care burden than other caregivers. No study has explored the effect of socioeconomic status (SES) on the intensity of informal care provided by married female caregivers in China. The purpose of this study is to empirically examine how the SES of married female caregivers affects the intensity of the informal care they provide for their parents/parents-in-law in China.

**Methods:**

The data for this study were drawn from 8 waves of the China Health and Nutrition Survey (CHNS). The respondents were married women whose parents/parents-in-law needed care and lived in the same city as them. SES was defined based on four indicators: education, economic status, employment status, and *hukou* (China’s household registration system). Informal caregivers were divided into three categories: non-caregivers (0 h/week), low-intensity caregivers (less than 10 h/week), and high-intensity caregivers (10 h/week and above). Multinomial logistic regression analysis was used to examine the relation between SES and the likelihood of a low- and high-intensity caregiving among married female caregivers, adjusting for age, family characteristics and survey wave.

**Results:**

Of the 2661 respondents, high-intensity and low-intensity caregivers accounted for 16.35 and 21.27%, respectively. The multinomial logistic regression results showed that the likelihood of being a high-intensity caregiver versus (vs. a non-caregiver) increased as the caregiver’s educational attainment increased (*p* < 0.05), and that high economic status was related to the likelihood of being a high-intensity caregiver, but this relationship was only significant at the 10% level. Urban females were 1.34 times more likely than their rural counterparts to provide low-intensity care vs. no care (*p* < 0.05) and were 1.33 times more likely to provide high-intensity care vs. no care (*p* < 0.05). Employed females were 1.25 times more likely than those unemployed females to provide low-intensity care vs. no care (*p* < 0.05).

**Conclusions:**

Differences in SES were found between high-intensity caregivers and low-intensity caregivers. Women with high educational attainment and urban *hukou* were more likely to provide high-intensity informal care, and women who were employed and had urban *hukou* were more likely to provide low-intensity care.

**Supplementary Information:**

The online version contains supplementary material available at 10.1186/s12877-021-02094-0.

## Background

With the rapid economic growth, longer life expectancy, and declining fertility rates, China has been considered an ageing society since 1999 [1]. The United Nations defines an ageing society as a country or region in which 10% of the population is aged over 60 or 7% is aged over 65 [2]. It took 115 years for France, 85 years for Switzerland, 80 years for the United Kingdom, 60 years for the United States, and just 18 years for China to transition to an ageing society [3]. In China, the National Bureau of Statistics generally defines older people as those over 65 years [4]. According to the latest data from the World Bank, the number of people aged 65 years and above had reached 160 million (11.5% of the population) in 2019 in China, and this number is estimated to increase to 239 million (16.9%) by 2030. Meanwhile, the number of older people in need of care is also increasing, which will inevitably bring serious challenges to long-term care services in China [5].

The World Health Organization (WHO) defines long-term care as a systematic activity, undertaken by informal caregivers (family members, friends or neighbours) or formal caregivers (such as professional medical institutions), to ensure that individuals incapable of fully caring for themselves can maintain a high quality of life [6]. In China, formal long-term care still in its infancy, there were only 2.7 registered nurses per thousand people and 29.1 beds provided by nursing homes per thousand older people by the end of 2018 [7]. China is confronting unprecedented shortages in formal caregiving provision and care for older people will still be based mainly on informal care for a long time in the future [8].

In China, influenced by both traditional gender norms and Confucian ideals of filial piety, adult daughters (or daughters-in-law) usually provide daily help for their parents (or parents-in-law), while sons are more likely to provide intermittent assistance such as home repair, financial management [9–12]. In addition, females (daughters or daughters-in-law) may be more vulnerable than males—females have a risk of lower income, lower educational attainment, and limited opportunities to access resources, and the majority of informal care is provided by females [9–11, 13]. According to the data from the China Statistics Bureau [14], females are 2.8 and 1.3 times more likely than males to do housework and to care for older people, respectively. Married females, in particular, deserve more attention because this group have to consider not only the care of their parents, but also the responsibilities of taking care of their parents-in-laws, as well as the pressures of taking care of their own families and working their jobs. Socioeconomic status (SES), a comprehensive indicator measuring the position of individuals within systems of inequality in the society [15], plays a key role in the informal care process. It is important to understand how the SES of married female caregivers affects the amount of informal care they provide for their parents/parents-in-law in China. The SES disparity among married female caregivers and its effects on the distribution of informal care burdens should be considered in the design of welfare programmes for fair contribution and compensation of informal care in society, and deserve policy attention.

Previous studies on the association between SES of caregivers and informal caregiving mainly focused on high-income countries and have shown mixed results. Most studies have indicated that caregivers with low SES may provide more informal caregiving than those with high SES [16–21]. By contrast, a study comparing informal caregiving among Hungary, Poland and Slovenia found that a higher education level was associated with a higher probability of being a caregiver in Poland, but there was no significant difference by educational level in Hungary or Slovenia [22]. However, the different contexts in low-and middle-income countries (LMICs) make these results from high-income countries be less generalizable to LMICs. Few studies have investigated the link between SES and informal care in LMICs, and only one study using World Health Survey data from 48 LMICs found that most informal caregivers had higher SES than non-caregivers [23]. Nevertheless, evidence from China, especially from studies on the SES of caregivers in informal care intensity in China among daughters/daughters-in-law, remains lacking. We found no literature from other countries with similar cultural values, such as other Asian countries, and the existing studies have mostly focused on comparing the demographics and SES of caregivers and non-caregivers, making little distinction between the different intensities of informal care [17]. High-intensity caregivers differ significantly from low-intensity caregivers in a variety of ways, from their demographic characteristics and their responsibilities in caregiving to the impact of caregiving on those they care for and also themselves. With increasing caregiving intensity, the percentage of caregivers reporting fair or poor general health also increases [24, 25]. High-intensity care providers may be particularly vulnerable to higher emotional stress, economic stress, health issues, and a lower quality of life [26–28]. Therefore, it is necessary to classify different caregiving intensities to fully understand how SES affects the provision of informal care among married females in China.

The objectives of the present study were to empirically examine the association between the SES of married female caregivers and informal caregiving for their elderly parents when needed in China. We hypothesized that being a married female caregiver with high SES, assessed based on four indicators (i.e., educational attainment, *hukou*, household income, and employment status), may be associated with high-intensity informal care.

## Methods

### Study design and participants

Data for this study were extracted from the China Health and Nutrition Survey (CHNS), a large-scale, longitudinal study in both urban and rural areas in nine provinces: Liaoning, Shandong, Heilongjiang, Henan, Jiangsu, Hubei, Hunan, Guizhou, and Guangxi. The CHNS was established as a joint project of the University of North Carolina at Chapel Hill and the Chinese Center for Disease Control and Prevention, and the goal is to determine how the social, economic, and demographic changes in China affect health and health behaviors across the life cycle [29]. The original survey launched in 1989 used a multistage random-cluster sampling process to select a sample from 72 counties in nine provinces in China [29], and nine additional surveys were conducted in 1991, 1993, 1997, 2000, 2004, 2006, 2009, 2011, and 2015. In this study, we pooled eight waves of data from 1993 to 2015, starting in 1993 because *hukou* status information has been incorporated since this wave [30]. Although the data were collected from a panel of individuals, the proportion of newly added individuals in each wave was over approximately 80% (for details, see Appendix Table S2). Thus, we analysed the data as eight waves of repeated cross-sections to avoid treating the panel members as a cohort since the age of the panel changed over time, and we used the clustering robust standard error to avoid individual autocorrelation.

The respondents were restricted to married women with at least one living parent or parent-in-law with care needs. Of the original 20,819 married female respondents, 3182 participants meeting this inclusion criterion (Respondents who provided an affirmative answer to the question “Does your parent/parent-in-law need to be taken care of in daily life and shopping?” were classified as having an elderly parent with care needs) were included. Then, 425 participants were excluded due to incomplete data, 16 women who did not live in the same city/county as their parents/parents-in-law who needed care were excluded to eliminate the impact of living distance, and 80 widowed and divorced individuals were excluded. Finally, 2661 respondents with complete data were included in our analysis.

### Outcome variable

In this study, we defined the informal care intensity provided by daughters or daughters-in-law as the dependent variable. Weekly hours of informal care were estimated based on the survey responses to the following question: “During the past week, how much time did you spend taking care of your parents or parents-in-law?” Following the current literature, we categorized the intensity of informal caregiving into three categories: non-caregivers (0 h/week of caregiving), low-intensity caregivers (less than 10 h/week of caregiving), and high-intensity caregivers (10 h/week of caregiving and above) [9, 31].

### Independent variables

The key explanatory variable in this study was socioeconomic status (SES). SES conventionally includes three indicators of educational attainment, household income, and employment status. Some researchers have indicated that *hukou* status should be included in SES, as it dictates the social benefits a person receives in China [32–34]. The *hukou* system (also called the household registration system) of China classifies all residents into rural and urban holders. Compared with urban *hukou* holders, those with a rural *hukou* may have lower education, fewer job opportunities, lower access to health benefits as well as poorer living conditions [30]. Thus, we defined respondents with urban *hukou* as having high SES and receiving higher benefits, than their rural counterparts. Therefore, this study used educational attainment (illiteracy, primary school degree, junior high school degree, high school degree, or university degree or above); economic status (net household income per year), employment status (employed or unemployed), and *hukou* status (urban or rural) to measure the women’s SES. The control variables included age, number of siblings, number of care recipient, age of care recipients (only information about whether they were older than 50 was available in the CHNS database), and survey wave. We chose all the independent variables based on previous studies [9, 17] on the determinants of informal care and the behavioural model of care service utilization proposed by Anderson and Newman [35]. The Anderson model divided potential determinants of informal care into three factors: need factor characteristics, enabling resources and actual need for care. Predisposing characteristics mainly included the number of care recipient and the age of care recipients and the age of the care recipient. Enabling characteristics mainly included caregiver age, education, number of siblings, etc. The survey wave variable was controlled to capture the time variation.

### Statistical analysis

We compared the SES of the caregivers with different informal care intensities using one-way analysis of variance (ANOVA) and chi-square tests as appropriate. Multinomial logistic (MNL) estimations were performed, using informal care intensity as the dependent variable and adjusting for the care recipient’s age and the survey wave. Relative risk ratios (RRRs) for different SES variables were computed. We also tested the independence of irrelevant alternatives (IIA) by using the Hausman-McFadden test to avoid inconsistent and IIA non-compliant parameter estimates [36]. We used STATA 14.2 (StataCorp, College Station, TX, USA) for all analyses.

## Results

### Descriptive results

Table 1 provides the summary statistics of the individual characteristics by level of caregiving intensity using the pooled sample of the 1993–2015 waves. The average age of the respondents was 41 years old, and the total number of respondents whose parents or parents-in-law needed to be cared for was 2661, of whom 1660 (62.38%) did not provide informal care. The proportion of high-intensity caregivers (16.35%) was lower than low-intensity caregivers (21.27%). Using Chi-square tests or ANOVA analysis, we found statistically significant differences in the four indicators of SES: educational attainment, *hukou* status, household income, and employment status. In terms of educational attainment, 16.78% of high-intensity and 11.48% of low-intensity caregivers had a university degree or above, while a lower proportion of those who did not provide care at all had a university degree (8.49%). Regarding *hukou* status, a higher proportion of high-intensity caregivers (50.11%) than low-intensity caregivers (46.47%) were urban *hukou* holders. The economic status of high-intensity caregivers (48,195 yuan/year) was higher than that of low-intensity caregivers (39,385 yuan/rear), and the economic status of low-intensity caregivers was higher than that of non-caregivers (34,229 yuan/year). We calculated the average care intensity (total care hours/parent numbers) and the results indicated that the average care intensity of high-intensity caregivers was higher than that of low-intensity caregivers (*p* < 0.001). Table 1 also indicates that there were statistically significant differences in age, number of siblings, number of parents, and wave at the 5% level.

**Table 1:** Descriptive statistics of the socioeconomic characteristics of women providing different intensities of informal care in China

Characteristic	All n (%)	Non-caregiver n (%)	Low-intensity caregiver n (%)	High-intensity caregiver n (%)	P-value
Total	2661	1660 (62.38)	566 (21.27)	435 (16.35)	
*Socioeconomic status*					
Educational attainment					< 0.001
Illiteracy	638 (23.98)	435 (26.20)	129 (22.79)	74 (17.01)	
Primary school degree	510 (19.17)	343 (20.66)	91 (16.08)	76 (17.47)	
Junior school degree	864 (32.47)	542 (32.65)	190 (33.57)	132 (30.34)	
High school degree	370 (13.90)	199 (11.99)	91 (16.08)	80 (18.39)	
University or above	279 (10.48)	141 (8.49)	65 (11.48)	73 (16.78)	
*Hukou* status					< 0.001
Rural	1561 (58.66)	1041 (62.71)	303 (53.53)	217 (49.89)	
Urban	1100 (41.34)	619 (37.29)	263 (46.47)	218 (50.11)	
Inflation-adjusted total annual household income, mean	37,733.288 (49,881.883)	34,228.794 (39,162.028)	39,384.547 (46,397.966)	48,195.006 (80,188.047)	< 0.001
Employment status					0.026
Unemployed	642 (25.72)	396 (25.47)	121 (22.79)	125 (30.49)	
Employed	1854 (74.28)	1159 (74.53)	410 (77.21)	285 (69.51)	
*Demographic characteristics*					
Age, mean (SD)	41.09 (7.03)	40.75 (7.24)	41.94 (6.41)	41.28 (6.89)	0.002
Number of siblings	2.95 (1.66)	2.89 (1.71)	2.98 (1.63)	3.14 (1.51)	0.017
Number of care recipient					0.003
1	1937 (72.79)	1244 (74.94)	411 (72.61)	282 (64.83)	
2	527 (19.80)	301 (18.13)	113 (19.96)	113 (25.98)	
3	126 (4.74)	69 (4.16)	30 (5.30)	27 (6.21)	
4	71 (2.67)	46 (2.77)	12 (2.12)	13 (2.99)	
Average care intensity (total care hours / parent numbers)	4.57 (13.42)	0	3.34 (2.20)	23.59 (25.55)	< 0.001
Age of care recipient					0.002
<50	51 (1.92)	44 (2.65)	5 (0.88)	2 (0.46)	
≥ 50	2610 (98.08)	1616 (97.35)	561 (99.12)	433 (99.54)	
Wave, n (%)					< 0.001
1993	258 (9.70)	183 (11.02)	52 (9.19)	23 (5.29)	
1997	288 (10.82)	196 (11.81)	56 (9.89)	36 (8.28)	
2000	345 (12.97)	226 (13.61)	72 (12.72)	47 (10.80)	
2004	411 (15.45)	269 (16.20)	82 (14.49)	60 (13.79)	
2006	343 (12.89)	229 (13.80)	56 (9.89)	58 (13.33)	
2009	274 (10.30)	164 (9.88)	60 (10.60)	50 (11.49)	
2011	365 (13.72)	201 (12.11)	90 (15.90)	74 (17.01)	
2015	377 (14.17)	192 (11.57)	98 (17.31)	87 (20.00)	

Note: χ^2^ tests were conducted with the categorical variables, and analysis of variance was conducted with the continuous variables; SD referes to the standard deviation

### Multinomial logistic regression analyses

Table 2 presents the multinomial logistic regression results for the SES of respondents, with non-caregivers defined as the reference group. We found that those with higher educational attainment and economic status were more likely to be high-intensity caregivers, and that those who were employed status were more likely to be low-intensity caregivers. Additionally, respondents with urban *hukou* were 1.34 times more likely to be low-intensity caregivers (*p* < 0.05), and 1.33 times more likely to be high-intensity caregivers than those with rural *hukou* (*p* < 0.05). Regarding employment status, employed respondents were 1.25 times more likely than unemployed females to be a low-intensity caregiver versus non-caregiver (*p* < 0.05). In terms of economic status, high income was related to the likelihood of being a high-intensity caregiver, but this relationship was significant only at the 10% level.

**Table 2:** Socioeconomic status of women providing different intensities of informal care: results of the multinomial logistic regression using the pooled CHNS data from 1993 to 2015 (*n* = 2661)

Variables	Low-intensity caregiver RRR (SE)	High-intensity caregiver RRR (SE)
*Socioeconomic status*		
Educational attainment		
Primary school degree	0.92 (0.15)	1.27 (0.23)
Junior school degree	1.15 (0.17)	1.20 (0.20)
High school degree	1.32* (0.25)	1.86*** (0.37)
University degree or above	1.16 (0.25)	1.96*** (0.43)
Economic status	1.01 (0.01)	1.01* (0.01)
Employment status: Employed	1.25** (0.14)	0.89 (0.11)
*Hukou* status: Urban	1.34** (0.16)	1.33** (0.17)
*Control variables*		
Age	1.02*** (0.01)	1.00 (0.01)
Number of siblings	0.95 (0.07)	0.97 (0.07)
Number of care recipients		
2	1.07 (0.14)	1.58*** (0.21)
3	1.19 (0.28)	1.54* (0.36)
4	0.66 (0.22)	0.95 (0.32)
Age of care recipient: ≥50	2.71** (1.29)	4.17* (3.16)
Wave		
1997	1.00 (0.22)	1.46 (0.42)
2000	1.35 (0.46)	1.84 (0.73)
2004	1.21 (0.41)	1.78 (0.70)
2006	0.94 (0.33)	1.94* (0.76)
2009	1.45 (0.52)	2.27** (0.91)
2011	1.66 (0.57)	2.40** (0.92)
2015	1.95** (0.63)	2.95*** (1.10)
Constant	0.03*** (0.02)	0.02*** (0.02)

Note: *** *p* < 0.01, ** *p* < 0.05,* *p* < 0.1;

*RRR* Relative risk ratio;

Robust standard errors are reported in parentheses;

Reference: illiterate (educational attainment), unemployed (employment status), rural (*hukou* status), Age < 50 (care recipient), 1 (number of parents who need cared for), 1993 (wave)

In Table 2, we also present the results of the control variables included in the analysis. When the age of the care recipients was over 50, the likelihood of being a high-intensity caregiver was 4.17 times higher than that of being a non-caregiver (*p* < 0.1*)*, and the likelihood of being a low-intensity caregiver was approximately 2.71 times higher (*p* < 0.05) than that of being a non-caregiver. No statistical significance was found based on the number of siblings. However, the respondents who had two parents needing care were 1.58 times more likely to be high-intensity caregivers than those who had only one parent needing care (*p* < 0.01). The results from the time trend analyses suggest a significant upward trend in high-intensity care. The relative risk ratio increased year by year since 2006 and reached 2.95 in 2015, which indicates that the respondents were increasingly likely to be high-intensity caregivers since 2006.

Table 3 shows the Hausman test results, which indicate that none of the three options would reject the IIA assumption (see Table 3). Spearman coefficients were used to test the correlation between SES variables (see Appendix Table S1).

**Table 3:** Hausman test results of the IIA

Omitted	Chi2	df	*P* > Chi2	evidence
0	8.457	10	0.584	for Ho
1	9.601	10	0.476	for Ho
2	8.446	10	0.585	for Ho

## Discussion

In this study, 62.38% of the participants whose parents or parents-in-law needed to be taken care of in daily life and shopping, did not provide informal care. We added the number of siblings of caregivers as a control variable to eliminate the impact of care provided by siblings, and the regression results show that the number of siblings had no statistical significance. Regarding the impact of formal care, formal care is not the first preference for older adults who need care in China. Previous studies have shown that formal care is a supplement rather than a substitute for informal care among older Chinese adults [37, 38]. In most cases, formal care is used only when adult children do not have time to provide informal care [37]. Moreover, Chinese people attach great importance to the concept of family affection, and informal care given by adult children can provide the necessary emotional support for older people [39]. Thus, even if a small proportion of formal care exists, informal care remains indispensable and cannot be replaced by formal care in China. Understanding the determinants of informal care is crucial in designing long-term care financing programmes.

SES is one key determinant affecting both caregivers and care-recipients in the caregiving context. It is crucial to develop targeting measures by analysing the SES of female caregivers, to design welfare programmes for informal care in China. To our knowledge, this is the first study to investigate the SES of married females who provide informal care for their parents/parents-in-law in need of care in China, and this study contributes to the literature on the ‘supply side’ of informal care. However, what we should highlight in the study is that this sample included only married women, so we need to be careful when extrapolating our findings. One of the key conclusions drawn from our findings is that high-SES females were more likely to provide informal care for their older parents compared with low-SES females, which is in agreement with the study of 48 LMICs by Louis et al. [40]. Specifically, those with high educational attainment, high economic status and urban *hukou* were more likely to provide high-intensity care, and females who were employed and had urban *hukou* were more likely to provide low-intensity care.

The current study showed that females with high educational attainment were more likely to be high-intensity caregivers, but this education effect was not found to be statistically significant in low-intensity informal caregivers. This finding is in agreement with the conclusion of Petra et al. that a higher educational level was associated with a higher probability of being a caregiver in Poland, although the authors did not focus on females [22]. One important reason for this finding might be due to the “feedback theory” proposed by Xiaotong Fei [41]. This “feedback theory” suggested that children have the responsibility to support their parents to repay for their upbringing and education. Education has long been an important factor in social and personal development, and attaining higher education requires greater parental and household investment. Those who have attained higher education may view caregiving as a way to provide for their parents and repay them for their investment [42]. In addition, females with high educational levels tend to be motivated by traditional Chinese culture, according to which it is a virtue to support one’s parents, especially when they are in need of care [43, 44].

Surprisingly, the employed women in this study were found to be more inclined to provide low-intensity informal care, whereas this association was not statistically significant for high-intensity informal care. To further explain this phenomenon, we performed an in-depth analysis to examine the correlation of the different SES (see Appendix Table S1). We found a positive correlation between education, income, and *hukou*, while employment status was only negatively correlated with *hukou*. Based on this result, we speculate that there may be a selection effect involved, especially for those with low SES. In recent decades, awareness of work and economic participation has increased [45]; thus, low-SES women also have to find a job for their basic livelihoods. It may be that when women are weighing decisions to find a job versus to take care of their parents or parents-in-law who need care, they will consider the intensity of the care their parents need. If the parents or parents-in-law only need a low-intensity care, they may prefer to work. However, high-intensity care requires a certain amount of time and effort, and thus high-intensity caregivers have to spend much time providing informal care for their parents or parents-in-law, regardless of their employment status.

Consistent with previous studies, we also found that women with urban *hukou* were more likely to provide informal care than those with rural *hukou* [40, 46]. Because we excluded those who did not live in the same city or county as their parents, the impact of living distance for rural respondents moving to urban areas for better job opportunities was eliminated. As we mentioned in the introduction, individuals with urban *hukou* receive higher benefits than their rural counterparts. Thus, one of the potential explanations for the urban-rural disparity observed in this study may be that rural *hukou* holders have limited time to care for their parents due to livelihood or lack the awareness of care for their parents who are in need. In addition, compared with rural women, urban women have a higher educational level, and higher education was found to be associated with more informal caregiving. The association is also demonstrated in the Appendix Table S1.

A negative association between caregiver SES and informal care has been found in some previous studies, which are inconsistent with the current study. However, these studies mainly focused on high-income countries. For example, in Germany, those who provided high-intensity informal caregiving tended to have low educational attainment and unemployment status [18]. A study in Belgium found that women who were not formally employed were more likely to be informal caregivers [21]. Another study in Japan found that women with lower education were more likely to be informal caregivers [17]. Agree and Glaser [47] suggested that SES may have different effects across societies and countries. One of the explanations for the inconsistent results may be due to the difference in the long-term care system between China and other countries. In high-income countries, economic and social development is ahead of population ageing, which allows for the establishment of a good economic and social foundation to address population ageing. Consequently, as the United States, Germany, Japan and other high-income countries have gradually established a relatively complete long-term care service system and social services in conjunction with health services to provide care for dependent older people, but in many LMICs, social services do not exist or remain dysfunctional [48]. Thus, in the abovementioned high-income countries, women with higher SES have a greater capacity to pay for formal institutional care for their older parents instead of informal care. Although the study in Poland reported findings consistent with our findings [22], this consistency may be due to the lack of a public insurance fund for long-term care for older people. In contrast, in China and many other LMICs, long-term care system are still in their infancy, and informal care remains dominant. Women with low SES may be less able to provide care for their older parents, which may be because they have to prioritize paid jobs due to financial needs and, for rural *hukou* holder, lower levels of social security.

As seen from trends over time, the number of people providing high-intensity informal care has been increasing over the last two decades. There are two possible reasons for this finding. First, according to the National Bureau of Statistics [4], the proportion of the population over 65 years has been rapidly increasing since 2000, which has generated a higher need for high-intensity care for elderly people. However, the Chinese government has not been well prepared for coping with this challenge, and the supply of formal care is not sufficient. Instead, an increase in informal high-intensity care has been observed. Second, even though we used the clustering robust standard error to avoid individual autocorrelation across eight waves of the data, the increase in high-intensity care in recent waves might be partly because the care recipients were older with each wave, and the need for high-intensity care increased.

The present study has several limitations. First, since the sample only includes married women aged 52 and below who participated in the supplementary survey on intergenerational linkages to parents, we may have underestimated the results of informal caregiving. Second, owing to the cross-sectional nature of the study, we cannot exclude the possibility of reverse causation. For example, the intensity of informal caregiving could conversely affect the employment status or income. Finally, the respondents were not asked what kind of care was being provided or whether other forms of caregiving (such as spousal care or paid formal care) were being used, and information on the specific age of the care recipient was not available in the database, making the analysis less comprehensive.

## Conclusions

In this study, we empirically examined the association between the SES of married female caregivers and their informal caregiving for their older parents in need of care in China. We found differences in SES between high-intensity informal female caregivers and low-intensity female caregivers. Women with high educational attainment and urban *hukou* are more likely to provide high-intensity informal care, and women who are employed and with urban *hukou* are more likely to provide low-intensity care. These findings suggest that low-SES women may be less able to provide care for their older parents, which may be because they have to prioritize working a paid job due to financial needs and, for rural *hukou* holders, lower levels of social security. Therefore, policy makers should consider the opportunity costs of informal care provision among married females when making policy recommendations about the design and funding of public long-term care programmes in the future.

## Supplementary Information

**Additional file 1.**

## Data Availability

The datasets are open to all of the potential users online. [http://www.cpc.unc.edu/projects/China].

## Abbreviations

SES: Socioeconomic status
CHNS: China Health and Nutrition Survey
RRR: Relative risk ratio
