# Supplementary material for: The effect of socioeconomic status on informal caregiving for parents among adult married females: evidence from China
Source: BMC Geriatr. 2021 Mar 6;21:164. doi: 10.1186/s12877-021-02094-0 (PMC7937217; doi:10.1186/s12877-021-02094-0)
Supplement: Supplementary file 1 — Additional file 1. [file 12877_2021_2094_MOESM1_ESM.docx]

**Table S1** The correlations matrix of socioeconomic status among caregivers (N = 2661)

|  | Education | Income | Employment | Hukou |
| --- | --- | --- | --- | --- |
| Education | 1.0000 |  |  |  |
| Income | 0.2258  (0.0000) | 1.0000 |  |  |
| Employment | -0.0079  (0.7690) | 0.0276  (0.1674) | 1.0000 |  |
| Hukou | 0.4510  (0.0000) | 0.1093  (0.0000) | -0.0884  (0.0000) | 1.0000 |

Note: *p*-value was reported in the parentheses.

**Table S2** The number of follow-up and new-added respondents in each wave from 1993 to 2015

| Wave | N | ≥ 4-wave follow-up  n (%) | 3-wave follow-up  n (%) | 2-wave follow-up  n (%) | New-added  n (%) |
| --- | --- | --- | --- | --- | --- |
| 1993 | 258 | 0 | 0 | 0 | 258 |
| 1997 | 288 | 0 | 0 | 32 (11.11) | 256 (88.89) |
| 2000 | 345 | 0 | 7 (2.03) | 37 (10.72) | 301 (87.25) |
| 2004 | 411 | 0 | 10 (2.43) | 68 (16.55) | 333 (81.02) |
| 2006 | 343 | 1 (0.29) | 19 (5.54) | 71 (20.70) | 252 (73.47) |
| 2009 | 274 | 3 (1.09) | 16 (5.84) | 49 (17.88) | 206 (75.18) |
| 2011 | 365 | 7 (1.92) | 21 (5.75) | 30 (8.22) | 307 (84.11) |
| 2015 | 377 | 9 (2.39) | 0 | 62 (16.45) | 306 (81.17) |
